# Effect of 4 Weeks of Anti-Gravity Treadmill Training on Isokinetic Muscle Strength and Muscle Activity in Adults Patients with a Femoral Fracture: A Randomized Controlled Trial

**DOI:** 10.3390/ijerph17228572

**Published:** 2020-11-19

**Authors:** Pyeongon Kim, Haneul Lee, Wonho Choi, Sangmi Jung

**Affiliations:** 1 Department of Physical Therapy, Ansan Hospital, Ansan 15324, Korea; kpo12@naver.com; 2 Department of Physical Therapy, Gachon University, Incheon 21936, Korea; leehaneul84@gachon.ac.kr; 3 Department of Occupational Therapy, SangGi Youngseo College, Wonju 26339, Korea

**Keywords:** electromyography, exercise, femoral fracture, muscle strength, rehabilitation

## Abstract

This study aimed to identify the effect of anti-gravity treadmill training on isokinetic lower-limb muscle strength and muscle activities in patients surgically treated for a hip fracture. A total of 34 participants were randomly assigned into two groups: anti-gravity treadmill training group (*n* = 17) and control group (*n* = 17). The isokinetic muscle strength and endurance of hip flexor and extensor and the activities of the vastus lateralis (VL), vastus medialis (VM), gluteus maximus (GM), and gluteus medialis (Gm) muscles were measured before and after 4 weeks of the interventions. Significant improvements were observed in isokinetic muscle strength and endurance of hip flexors and extensors in both groups (*p* < 0.05); however, no significant differences were observed between the groups (*p* > 0.05) except for muscle strength of the hip extensor (*d* = 0.78, *p* = 0029). Statistically significant increases in the muscle activity of VL, VM, GM, and Gm were found before and after the intervention (*p* < 0.05), and significant differences in muscle activities of GM (*d* = 2.64, *p* < 0.001) and Gm (*d* = 2.59, *p* < 0.001) were observed between the groups. Our results indicate that both groups showed improvement in muscle strength, endurance, and activities after the intervention. Additionally, anti-gravity treadmill training improved significantly more muscle strength at 60°/s of the hip extensor and gluteus muscle activities than conventional therapy, which may be appropriate for patients with hip fracture surgery.

## 1. Introduction

Approximately 1.35 million people die annually in road traffic crashes, and millions more suffer from injuries caused by that [1]. Of patients injured in a road traffic crash, around 19–35.4% have femoral fractures [2]. Femoral fractures occur as a result of relatively strong forces in events such as falls and traffic accidents, because of the specificity of the anatomical structure of the femur bone. Recently, the frequency of fractures has increased owing to the increase in traffic accidents in modern society involving even younger adults [3]. Body weight is a crucial factor when standing upright or when walking, and the quadriceps muscle plays an important role in providing stability to the knee joint. The quadriceps, which is not able to bear weight after surgery, shows muscle loss and weakness, especially within the first 5 days, postoperatively. Postoperative functional limitations due to soft tissue defects including hip abductor, flexor muscles, and extensor cause limited gait function with decreased muscle weakness and pain [4,5]. The vastus medialis (VM) is physiologically the weakest muscle and is prone to muscular dystrophy. A weakened VM muscle poses a high risk of pain and injury around the knee joint owing to imbalance of the quadriceps muscles [6]. Therefore, isometric exercise of the hip flexor and extensor is an important component of rehabilitation for improving the patient’s walking performance, activities of daily living, functioning, pain, and muscular dystrophy [7]. In addition, weakened lower-limb muscles reduce walking speed and balance, whereas balanced development of the anti-gravity muscles of the core and pelvis enables safe and efficient lower-limb movement [8,9].

The postoperative rehabilitation program should be adjusted according to the degree of stability of the fracture; however, rehabilitation using a wheelchair, gait training with parallel bars, and gait training with a walking aid are commonly performed in progression. Elderly patients tend to voluntarily limit their weight bearing on the operated side, although it is possible to increase weight bearing to approximately 51% of the normal weight in the first week after surgery [10]. In addition, in gait training, strengthening of the quadriceps and hip abductors should be progressively performed to reduce disruptions and prevent falls [11]. 

An anti-gravity treadmill is a device that uses a special air pressure control system to alter the gravity felt in the lower extremities during walking training. This has an effect of reducing the patient’s weight to 80%, thus enabling walking and running exercises without 100% weight bearing. This device is also safe and effective for the rehabilitation of postoperative patients because of its precision control of up to 1% of the body weight, enabling rehabilitation without pain in patients with lower-limb injury [12]. The walking distance can be increased while maintaining normal walking, and walking and running activities can be performed without causing changes in the range of motion of the ankle and knee joints [13]. Anti-gravity treadmill training also effectively reduces the amount of impact on the knee when walking. A previous study has shown the effectiveness of this training method in early rehabilitation and in reducing the force transmitted to the knee by adjusting the gravity to 50% of the body weight [14]. Moreover, previous studies have reported that muscle strength exercise and aerobic exercise using an anti-gravity treadmill resulted in improved walking and dynamic balance, while maintaining the same kinetic movement as normal walking and reducing the amount of pressure on the musculoskeletal system [13,15,16]. Thus, anti-gravity treadmill training is an intervention for preventing quadriceps muscle atrophy and for muscle strengthening in patients with femoral fractures [17,18]. Further, it enables partial weight bearing at the initial stage, which may help provide stability for normal gait in the future.

Therefore, the purpose of the present study was to investigate the effect of a rehabilitation training program using an anti-gravity treadmill on the strength and activity of lower-extremity muscles in patients surgically treated for a hip fracture.

## 2. Materials and Methods

### 2.1. Ethical Approval

This study was approved by the Institutional Review Board of Gachon University (1044396-201902-HR-008-01). All participants signed a statement of informed consent before beginning the study.

### 2.2. Participants

A total of 34 patients who undergone femoral fracture surgery due to a femoral fracture participated in this study. Subjects were included if they were surgically (internal fixation or replacement arthroplasty) treated for femoral fracture and were able to do weight bearing on postoperative assessment. Patients with musculoskeletal impairment in the lower extremities except for a traumatic femoral fracture, such as pathologic fracture, knee joint ligament impairment, meniscal impairment, ankle joint impairment, hip joint and pelvic impairment and dislocation, and neurological impairment, were excluded. Furthermore, patients who underwent hip surgery for infection, arthritis, or avascular necrosis were excluded from the study. The general characteristics of the participants are described in Table 1.

### 2.3. Procedures

The 34 patients who met the criteria were randomly assigned into two groups using random numbers in Microsoft Excel 2010, and their general characteristics were recorded. The participants were randomly allocated into two groups: experimental group (*n* = 17) and control group (*n* = 17). This study was double blinded, and different investigators performed the randomization, outcome measurement, intervention (both anti-gravity treadmill training and conservative rehabilitation exercise), and statistical analysis to minimize potential biases.

The general characteristics of the participants, including age, sex, height, weight, body mass index, and onset date, were collected. The isokinetic muscle strength and muscle activity of the gluteus maximus (GM), gluteus medius (Gm), vastus lateralis (VL), and VM were measured at baseline. The experimental group underwent 20 min of anti-gravity treadmill training five times per week for 4 weeks. The control group underwent 20 min of conservative rehabilitation five times per week for 4 weeks. The isokinetic strength and activity of the four muscles were measured after 4 weeks of intervention in both groups.

### 2.4. Intervention

#### 2.4.1. Anti-Gravity Treadmill Training

An anti-gravity treadmill (version 1.20, model: Anti-gravity Treadmill M320/F320; Altern-G Inc., Fremont, CA, USA) uses the air pressure inside a chamber to counter the gravity in the internal state. Accordingly, the gravity load and weight inside the chamber are evenly reduced, allowing precise weight control. To walk in an anti-gravity environment, the patients wore special pants that prevent air from escaping. After a weight measurement, the patients performed anti-gravity treadmill training inside the chamber for 20 min (Table 2).

#### 2.4.2. Conventional Rehabilitation (Control Group)

The conventional rehabilitation program was based on the exercise methods of Vanessa [15] and William [16], with modified and secure protocols, and performed for 20 min at each session (Table 3).

### 2.5. Outcome Measurements

#### 2.5.1. Isokinetic Strength Measurement

The BTE Primus RS kinetic test evaluation device (BTE, Hanover, MD, USA) was used to measure isokinetic muscle strength. Muscle strength was measured at a speed of 60°/s, with a total of five flexions and extensions of the knee joint, and the maximum torque of the range of motion was measured. After measuring the maximum torque, a 1 min rest time was provided. Thereafter, 15 measurements at an angle velocity of 180°/s were performed for the evaluation of muscle endurance.

#### 2.5.2. Muscle Activities

Neuromuscular muscle activity was measured using four-channel electromyography (EMG) with Clinical Direct Transmission System(DTS) (Noraxon Inc, Scottsdale, AZ, USA). The measurement sites were marked and disinfected with alcohol before placing the electrodes. Before the experiment, maximal voluntary isometric contraction (MVIC) was measured in manual muscle test posture for 5 s, three times, and the average was used for the analysis. Each measurement had a 5 s rest time, and a 2 min rest time was provided between each muscle measurement. The electrodes were attached to four muscles in the participant’s dominant lower extremity according to the European Recommendations for Surface EMG/International Society of Electromyographic and Kinesiology protocol [19]. The collected surface EMG signals were processed using MR-XP 1.08 Master Edition DTS (Noraxon Inc., Scottsdale, AZ, USA). Raw data were analyzed through a filtering process by setting the band-pass filter to 20–400 Hz. After rectification, the 200 ms filtered root mean square was analyzed. 

To measure GM activity, electrodes were attached at the midpoint between the sacrum and greater trochanter of the femur in the prone position. To measure Gm activity, electrodes were attached at the midpoint between the iliac crest and greater trochanter of the femur in the side-lying position. To measure VM activity, electrodes were attached at the midpoint between the anterior line of the medial ligament of the knee joint and 4/5 of the anterior superior iliac spine in the sitting position with slight knee flexion. To measure VL activity, electrodes were attached at the lateral side of the femur, which is above the fifth finger point of the patella.

Measurement was performed in the sitting position without the foot touching the floor in order to measure the EMG signals of the maximum voluntary isokinetic contraction of the VM and VL. After instructing the participant to take a starting position by keeping the knee joint at 90° flexion, the participant extended the knee, resisting the investigator’s force at the upper part of the ankle joint and producing the MVIC. After instructing the participant to abduct the hip joint by 20° and perform external rotation by 10° in the side-lying position, the Gm was measured as the participant maximally abducted in that position, resisting the investigator’s inferior force at the ankle joint. GM was measured in the prone position with 90° of knee flexion. The participants extended the hip joint by resisting the investigator’s force at popliteal fossa [20]. 

### 2.6. Sample Size Estimation

G power 3.0.1 software (Heinrich Heine University Düsseldorf, Düsseldorf, Germany) was used to acquire the sample size. With our pilot study results, a total of 30 participants were estimated to be required with an effect size of f = 0.186, significance level of 0.05, power of 0.80, and correlation among rep measures of r = 0.768 when a clinically significant interaction was observed between time points (two events: pre and post) and two groups.

### 2.7. Statistical Analysis

Statistical analysis was performed using SPSS 25.0 software (IBM, Armonk, NY, USA) to calculate all measured values as means and standard deviations. The normal distribution of variables was examined using the Shapiro–Wilk test, and all outcome variables were found to be normally distributed. A 2 × 2 mixed factorial analysis of variance (ANOVA) was conducted to determine whether there was any interaction between group and time effects. An independent-sample t-test was performed to compare the differences in dependent variables at 4 weeks before the intervention between the two groups. Moreover, analysis of covariance (ANCOVA) was used to explore the influences of any independent variables over the dependent variables. The level of significance was set at α = 0.05.

## 3. Results

A total of 34 patients participated in this study (26 men and 8 women). No significant difference was found between the groups in the homogeneity test (*p* > 0.05).

### 3.1. Isokinetic Strength (Peak Torque)

#### 3.1.1. Isokinetic Muscle Strength at 60°/s

The hip extension speed of 60°/s before and after anti-gravity training in the experimental group showed a significant interaction between group and time (*p* = 0.029). For the hip flexion speed of 60°/s, no significant interaction between time and group was found (*p* = 0.444). The hip flexors did not show a significant difference between groups (*p* = 0.520); however, both groups showed significant improvement in hip flexors at 60°/s (*p* < 0.001 and <0.001, respectively).

#### 3.1.2. Isokinetic Muscle Strength (Muscle Endurance) at 180°/s

The extension and flexion speed of 180°/s showed no significant interaction between group and time (*p* = 0.244 and 0.286, respectively). After the interventions, hip extensor and hip flexor endurance in both groups was statistically improved (*p* < 0.001 and <0.001, respectively). However, no statistically significant difference in was found between groups (*p* = 0.343 and 0.429, respectively, Table 4 and Table 5).

### 3.2. Muscle Activity

No significant interaction was found in the muscle activities of VL and VM between group and time (*p* = 0.646 and 0.553, respectively). The muscle activities of VL and VM significantly increased after 4 weeks of intervention (*p* < 0.001 and 0.001, respectively); however, no statistical difference was found between groups (*p* = 0.325 and 0.835, respectively, Table 4 and Table 5). 

A significant interaction was observed in the muscle activities of GM and Gm between group and time (*p* < 0.001). Both groups showed significant increase on GM and Gm muscle activities before and after the intervention (*p* < 0.001 and <0.001, respectively). In addition, a significant group effect was found on GM and Gm muscle activities (*p* < 0.001 and 0.004, respectively, Table 4 and Table 5).

We also adjusted covariates including sex, age, and BMI baseline group differences in each variable but no differences were found after adjusting covariates. 

## 4. Discussion

Sudden and strong weight bearing to the lower extremities in the course of rehabilitation after surgery for a femoral fracture causes damage to the surgical site, leading to the possibility of rehabilitation failure. Elaborate and stable weight bearing in the operated region is considered a crucial point in rehabilitation after a hip fracture surgery [18]. Thus, the present study was intended to investigate the effectiveness of rehabilitation focused on weight bearing with an anti-gravity treadmill in patients who underwent hip fracture surgery due to traumatic hip fracture.

Previous studies have shown that anti-gravity treadmill training has a positive effect on knee muscle function in patients with meniscus rupture and knee osteoarthritis [16,21,22]. One study has shown that anti-gravity treadmill training improves spatiotemporal parameters, knee gait pattern, and muscle strength in patients with knee osteoarthritis, consequently improving the activities of daily living [16]. Another study found no significant difference in muscle activity between GM and Gm after non-weight bearing exercises in athletes with no orthopedic damage [23]. However, our study showed a significant group difference in GM and Gm muscle activities, which might be affected by pelvic stability. In particular, during the gait cycle, the GM at the initial contact of the gait cycle allows the position of the hip joint and pelvis to be dynamically stable and to move forward. The Gm muscle stabilizes pelvic tilt through eccentric contraction during the mid-stance of the gait cycle [24].

The significant difference in gluteus muscle activities between the two groups may be due to slow gait training with single-leg stand in the early stages of the anti-gravity treadmill rehabilitation protocol. Gm plays an important role in balancing the pelvis on the frontal plane when standing on a single leg during normal gait [25]. In addition, Gm forms twice the torque of its weight to provide stability to the pelvis and hip joints in the stance phase, and it was sufficient to increase muscle strength as the peak torque changes over 4 weeks. However, increased GM and Gm muscle activities are involved in the stability of the hip joint with single-leg stands during anti-gravity treadmill training rather than during the conventional rehabilitation. Therefore, the anti-gravity treadmill training program was designed to be secure through a quantified weight-bearing protocol and may have a positive effect on the rehabilitation of patients who have undergone hip fracture surgery due to traumatic hip fracture.

Rehabilitation is encouraged to reduce systemic complications caused by long-term immobilization and bed rest during the early stage of treatment, which is the main goal in hip fracture patients [26]. In this respect, the lack of significant differences in the isometric strength and muscle activity of the two groups demonstrates the effectiveness of both training types while emphasizing the importance of rehabilitation. However, anti-gravity treadmill training was superior to conventional rehabilitation in terms of hip stability because the single-leg stand resistance exercise showed high levels of muscle activity in the GM and Gm.

This study had some limitations. First, the training effect was difficult to objectify because the extent of injury to the femur was diverse. As the patients’ individual bone union and recovery rates were different, the results cannot be generalized; thus, the external validity is not high enough. Second, the subjects who participated in our study had a femoral fracture mostly from motor vehicle accident, therefore our subjects may not represent the entire population of hip fracture patients. Third, only changes in isokinetic strength (peak torque) were measured, and measurements of isokinetic strength (peak torque/body weight) and total work per unit weight were not obtained. Therefore, not all kinematics were considered. Future studies should investigate the effects on kinematics progress each week and for how long the anti-gravity treadmill training program should be performed in order to allow full weight-bearing training.

## 5. Conclusions

In conclusion, both groups showed improvement in muscle strength, endurance, and activities after the intervention. Additionally, anti-gravity treadmill training improved significantly more muscle strength at 60°/s of hip extensor and gluteus muscle activities than conventional therapy. Therefore, anti-gravity treadmill training compensates for the shortcomings of the conventional intervention used in rehabilitation and offers a rehabilitation protocol for a stable and effective gait in patients with a femoral fracture.

## Figures and Tables

**Table 1:** General characteristics of the participants (*n* = 34).

	EG (*n* = 17)	CG (*n* = 17)	*p*
Female sex, *n* (%)	4 (23.5%)	4 (23.5%)	1.000
Age (years)	48.82 ± 5.96	51.82 ± 5.91	0.150
Height (cm)	169.94 ± 8.21	168.88 ± 8.77	0.719
Weight (kg)	63.12 ± 5.13	66.12 ± 7.70	0.191
BMI (kg/m^2^)	21.89 ± 1.56	23.21 ± 2.57	0.077
Days after surgery	25.06 ± 3.56	23.76 ± 2.84	0.251
Cause of femoral fracture			
Motor vehicle accidents	14 (82.3%)	12 (70.6%)	0.419
Falls	3 (17.7%)	5 (29.4%)	
Type of fracture			
Proximal femoral fracture	3 (17.7%)	4 (23.5%)	0.523
Femoral shaft fracture	14 (82.3%)	12 (70.6%)	
Supracondylar femoral fracture	0 (0%)	1 (5.9%)	
Type of surgery			
Internal fixation	17 (100.0%)	15 (88.2%)	0.145
Replacement arthroplasty	0 (0%)	2 (11.8%)	

Values are expressed as mean ± standard deviation. Abbreviations: EG, experimental group (anti-gravity treadmill training); CG, control group (conventional rehabilitation); BMI, body mass index.

**Table 2:** Intervention procedure (anti-gravity treadmill program).

Period (weeks)	Gravity (%)	Velocity (mph)	Slope (°)	Time (min)
1	<25%	2.0–4.0	0	20
2	25–50%	4.0–6.0	0	20
3	50–75%	6.0–8.0	0	20
4	75–100%	8.0–10.0	0	20

**Table 3:** Conservative rehabilitation exercise.

	Exercise	Count/Set
1–2 weeks	Q-setting	12/3
	Hip adduction	
	Hip abduction	
	Lateral position hip hold exercise	
3–4 weeks	Heel slide exercise	12/3
	Straight leg raise	
	Prone and knee flexion position hip extension	
	Lateral position hip abduction	

**Table 4:** Isokinetic muscle strength and muscle activities before and after the intervention (*n*= 34).

Variables	Sources		F	*p*
60°/s Hip extension	Within subjects	Time	5171.684	<0.001
		Time × group	5.243	0.029
	Between subjects	Group	0.533	0.471
60°/s Hip flexion	Within subjects	Time	702.458	<0.001
		Time × group	0.599	0.444
	Between subjects	Group	0.424	0.520
180°/s Hip extension	Within subjects	Time	2528.780	<0.001
		Time × group	1.410	0.244
	Between subjects	Group	0.925	0.343
180°/s Hip flexion	Within subjects	Time	5344.682	<0.001
		Time × group	1.175	0.286
	Between subjects	Group	0.643	0.429
Vastus lateralis	Within subjects	Time	1238.600	<0.001
		Time × group	0.214	0.646
	Between subjects	Group	1.001	0.325
Vastus medialis	Within subjects	Time	417.725	<0.001
		Time × group	0.360	0.553
	Between subjects	Group	0.044	0.835
Gluteus maximus	Within subjects	Time	1275.711	<0.001
		Time × group	59.074	<0.001
	Between subjects	Group	21.169	<0.001
Gluteus medius	Within subjects	Time	2622.864	<0.001
		Time × group	58.108	<0.001
	Between subjects	Group	9.730	0.004

**Table 5:** Isokinetic muscle strength and muscle activities in hip fracture patients before and after the intervention (n= 34).

		EG (*n* = 17)	CG (*n* = 17)	*p* *	E
60°/s Hip extension	Pre	78.41 ± 9.36	77.88 ± 12.69	0.891	
	Post	144.18 ± 9.88	139.59 ± 9.92	0.186	
	DIFF	65.76 ± 5.29	61.71 ± 5.05	0.029	0.783
60°/s Hip flexion	Pre	38.41 ± 4.72	39.35 ± 9.66	0.721	
	Post	76.53 ± 10.44	79.76 ± 14.15	0.454	
	DIFF	38.12 ± 9.17	40.41 ± 8.01	0.444	0.266
180°/s Hip extension	Pre	56.59 ± 9.57	60.88 ± 13.04	0.282	
	Post	99.94 ± 7.14	102.29 ± 10.55	0.462	
	DIFF	42.35 ± 5.62	41.35 ± 4.08	0.245	0.204
180°/s Hip flexion	Pre	33.18 ± 8.97	36.65 ± 12.48	0.359	
	Post	69.41 ± 9.29	71.82 ± 11.98	0.516	
	DIFF	36.24 ± 2.31	35.18 ± 3.30	0.286	0.372
Vastus lateralis	Pre	129.53 ± 10.51	125.59 ± 10.65	0.286	
	Post	202.00 ± 21.64	196.18 ± 16.08	0.380	
	DIFF	72.47 ± 12.69	70.59 ± 10.95	0.647	0.159
Vastus medialis	Pre	121.41 ± 7.77	124.76 ± 11.99	0.341	
	Post	193.59 ± 25.51	192.82 ± 28.93	0.935	
	DIFF	72.18 ± 19.71	68.06 ± 20.29	0.553	0.206
Gluteus maximus	Pre	176.82 ± 12.40	174.41 ± 13.31	0.588	
	Post	301.06 ± 15.47	254.65 ± 25.80	<0.001	
	DIFF	124.24 ± 15.42	80.24 ± 17.87	<0.001	2.636
Gluteus medius	Pre	103.76 ± 13.23	101.53 ± 12.67	0.618	
	Post	211.82 ± 15.81	181.59 ± 21.21	<0.001	
	DIFF	108.06 ± 12.31	80.06 ± 9.07	<0.001	2.590

Abbreviations: EG, experimental group (anti-gravity treadmill training); CG, control group (conservative rehabilitation exercise); DIFF, difference; E, effect size. * *p*-value from the interaction between groups.
